# Diversity of Human Milk Oligosaccharides and Effects on Early Life Immune Development

**DOI:** 10.3389/fped.2018.00239

**Published:** 2018-09-10

**Authors:** Veronica Ayechu-Muruzabal, Arthur H. van Stigt, Marko Mank, Linette E. M. Willemsen, Bernd Stahl, Johan Garssen, Belinda van't Land

**Affiliations:** ^1^Division of Pharmacology, Utrecht Institute of Pharmaceutical Sciences, Faculty of Science, Utrecht University, Utrecht, Netherlands; ^2^Pediatric Immunology, Laboratory of Translational Immunology, The Wilhelmina Children's Hospital, University Medical Center, Utrecht, Netherlands; ^3^Department of Immunology and Department of Human Milk Research & Analytical Science, Danone Nutricia Research, Utrecht, Netherlands

**Keywords:** human milk oligosaccharides, mucosal immunity, tolerogenic dendritic cells, infections, early life nutrition

## Abstract

One of the well-known features of human milk, is the capacity to protect against the risk and impact of neonatal infections, as well as to influence the onset of allergic and metabolic disease manifestations. The major objective of this review is to provide a detailed overview regarding the role of human milk, more specifically the diversity in human milk oligosaccharides (HMOS), on early life immune development. Novel insights in immune modulatory effects of HMOS obtained by *in vitro* as well as *in vivo* studies, adds to the understanding on how early life nutrition may impact immune development. Extensive description and analysis of single HMOS contributing to the diversity within the composition provided during breastfeeding will be discussed with specific emphasis on immune development and the susceptibility to neonatal and childhood infections.

## The protective effect of breastfeeding against infections

It has been long noted that breastfeeding protects newborns against infections. Infant formula has been developed over many decades into adequate nutrition for those infants who cannot receive human milk. However, even modern infant formulas lack many components tailor made by each mother for the immune imprinting of her baby, such as specific antibodies (based on the immunologic history of the mother) and human milk oligosaccharides (HMOS) (based on the mother's specific genetic makeup regarding e.g., Lewis (Le) blood group and secretor (Se) status). Exclusive breastfeeding until the age of 4 months followed by partial breastfeeding is associated with a reduction in respiratory and gastrointestinal infectious diseases (1). For example, infants admitted to the hospital with Respiratory Syncytial Virus infection (RSV) are less likely to have been breastfed (2, 3). Similarly, infants who are not exclusively breastfed at 6–8 weeks of age, have a higher risk of hospitalization in early life in relation to a wide range of common infections (2). On the contrary, specific prebiotic oligosaccharides [like short chain galacto- and long chain fructo-oligosaccharides (scGOS/lcFOS)] are already added in a 9:1 ratio to plain infant formula and have been shown to reduce the development of atopic eczema and allergies as well as reduce the impact of pediatric infections (4, 5). Therefore, further optimization of infant nutrition when breastfeeding is unavailable is a principal factor required to further support immune development in early life.

The protective effect of human milk is postulated to be achieved by several mechanisms including the provision of pathogen specific maternal antibodies. This will provide the infant with pathogen specific protection during the first months of life, in which infant's own B cell development has not reached its full potential. Specifically, within the first years of life, the B cell repertoire matures upon encounter of pathogens, eventually providing a full range of protection against the recurrent pathogens. The immunoglobulins in human milk possess a broad range of pathogen specificity, which mirrors the maternal antigenic state. In addition, the concentration of soluble IgA (sIgA) are remarkably high and variable, and correlate to levels of IgG and IgM detected within different regions (6). At birth (for example via vaginal delivery as well as during breastfeeding) the neonate encounters a large variety of microorganisms which are determinant in the establishment of the microbiome in adult-life (7, 8). The initial colonization follows successive steps and is altered through the first year of life by diverse factors including genetic as well as environmental factors such as, introduction of oral feeding. Human milk was shown to stimulate healthy intestinal microbial diversity which includes colonization of several Bifidobacteria and Lactobaccillus species, which in turn, will result in the development of a balanced metabolic response (8, 9). In addition, human milk provides direct support to further development of the immune system in the neonate (10, 11).

The immune system of the neonates needs to adapt and respond to diverse stimuli encountered in early life. Immune homeostasis is determined by the cross-talk between exposure to the mucosal surfaces encompassing cross talk between epithelial cells and underlying immune cells (12). Human milk contains diverse factors like HMOS, milk epidermal growth factor or vitamin A, which contribute to the development of the neonatal mucosa and thus, to the promotion of the neonatal immune system by counterbalancing the deficiencies in early life. These variations are designated as poor IgA production, defective antimicrobial peptide secretion, lack of epithelial chemokine secretion as well as increased permeability among others (6, 10, 13).

Beyond functional components in human milk also the intestinal microbiota can help to further develop these aspects of the mucosal immune system (2). This emphasizes the relevance of a healthy intestinal microbiome diversity for adequate immune development in the first years of life (9). Next to the various classes of pathogen specific immunoglobulins in human milk, the presence of antimicrobial molecules, including specific non-digestible free carbohydrate structures and other molecules like glycoconjugates in breast milk have been shown to bind to pathogens (14). The nutrient source in early life, in particular the non-digestible human milk oligosaccharides (HMOS) in case of breastfeeding are of importance for healthy neonatal microbial colonization (7, 8, 12), immune development (15–19), as well as B cell development (20). This review aims to reveal the current state of knowledge regarding the immunomodulatory properties of HMOS and its unique complexity with differences in short chain as well as long chain structures. Recently some of these structures have become available via manufacturing procedures, and it might be considered to apply these in future generations of infant milk formula (21).

## Diversity of human milk oligosaccharide composition

**Figure 1:**
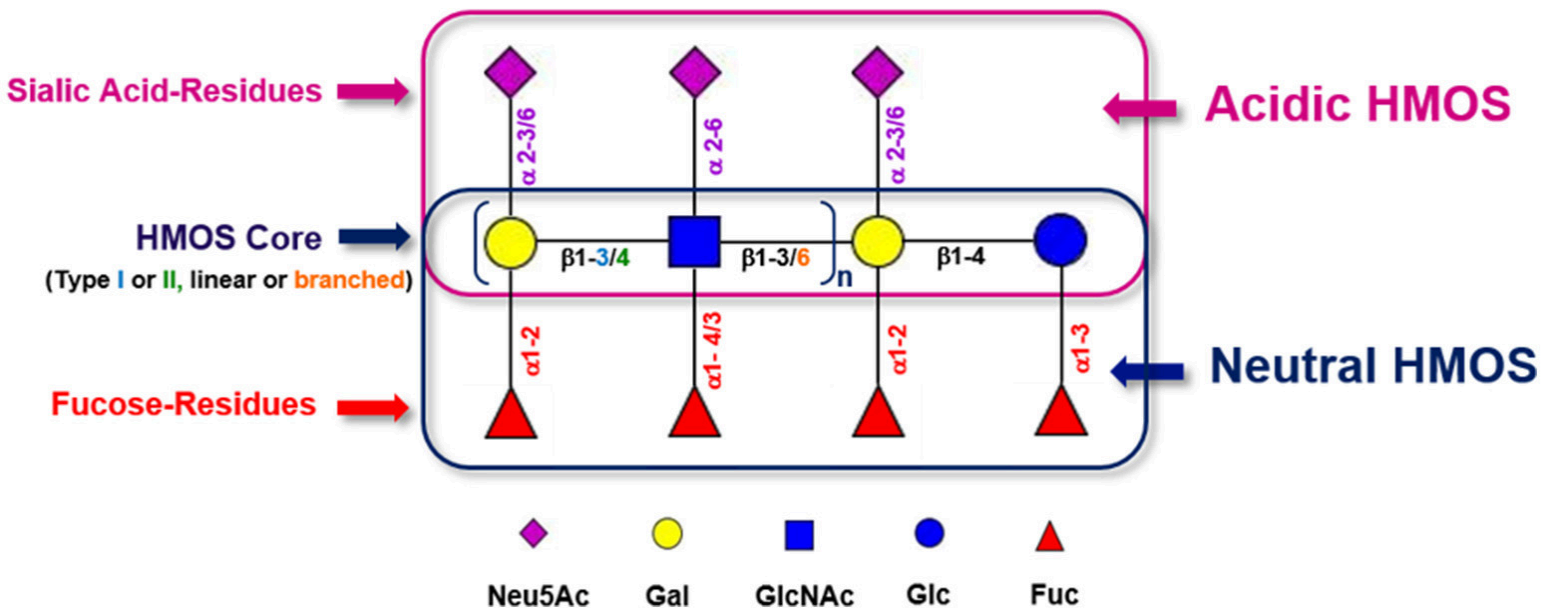
Generic building scheme of HMOS. Lactose and Type I (Gal (β1-3)GlcNAc-R) or Type II (Gal (β1-4)GlcNAc-R) HMOS core structures can be further extended linearly by adding additional Gal-GlcNAc building blocks to terminal Galactoses via β1-3 glycosidic linkages or via β1-6 glycosidic linkages. In the latter case, branching of the HMOS structure occurs. The (elongated) HMOS core structures can be further decorated with Fucoses (Fuc) and/or Sialic Acid (Neu5Ac) residues following distinct rules. Symbolic representation of monosaccharides according to CFG guidelines (29).

HMOS are the third most abundant class of biomolecules found in human milk after lactose and lipids, reaching between 5 and 20 g/L in mature human milk (22). Up to 1% of HMOS are absorbed in the gastrointestinal tract and found available in systemic circulation (23). This diversity and abundance is unique in humans and not seen in other mammals (24). The concentration of total HMOS is subjected to variations dependent on lactational stage (25), maternal nutrition (26), genetic predisposition (27) or even geographic localization and socioeconomic environment of milk donors (28). Although HMOS are composed out of only 5 different monosaccharides, the structural complexity of HMOS encountered in human milk is unique (21). The monosaccharides which are used as building blocks for HMOS are glucose (Glc), galactose (Gal), N-Acetyl-Glucosamine (GlcNAc), fucose (Fuc), and sialic acid (Neu5Ac). These single monosaccharides are conjugated via several linkage types (i.e., glycosidic bonds). With only a few exceptions, HMOS structures do follow a strict building plan (Figure 1). Each HMOS structure starts with a lactose unit “Gal (β1-4) Glc” which results from formation of a β1-4 glycosidic linkage between galactose and glucose catalyzed by the lactose synthase protein complex (30). Several tri-saccharides can be synthesized by appending either galactose or fucose to the reducing or non-reducing end of the lactose residue, which is performed through galactosyl- or fucosyl-transferase activity. Resulting components are e.g., 3′-galactosyllactose (Gal(β1-3)Gal(β1-4)Glc), 4′-galactosyllactose (Gal(β1-4)Gal(β1-4)Glc), 6′-galactosyllactose (Gal(β1-6)Gal(β1-4)Glc), 2′-FL (Fuc(α 1-2)Gal(β1-4)Glc), and 3-fucosyllactose (3-FL) (Gal(β1-4)[Fucα1-3]Glc). If sialic acids are connected to the non-reducing end of lactose via sialyl-transferases, 3′-sialyllactose (3′-SL; Neu5Ac (α 2-3)Gal(β1-4)Glc) and 6′-sialyllactose (6′-SL) (Neu5Ac(α 2-6)Gal(β1-4)Glc) are formed. Further elongation of lactose via the free 3-OH group of galactose can occur by addition of Gal (β1-x)GlcNAc units of either type I (Gal (β1-3)GlcNAc, Lacto-N-biose) or type II (Gal (β1-4)GlcNAc, N-Acetyllactosamine). Up to now, 19 different human milk oligosaccharide core structures have been described. These core structures may be linear or branched and can be further decorated with fucoses or sialic acid residues. Which indicates a myriad of different HMOS structures produced in the human mammary gland. The cellular localization of HMOS synthesis in the mammary gland epithelium is believed to be the Golgi apparatus.

Among other early life factors, the individual maternal genetic disposition has a huge influence on the HMOS profile of human milk. More specifically, the individual expression pattern of Lewis (Le) and Secretor (Se) gene alleles codes for different fucosyl-transferases (FUTs), as shown in Table 1 The activity of these FUTs can lead to fucosylation of lactose and various other human milk core structures as indicated.

**Table 1:** Relationship between maternal genotype and exemplified Le- or Se- related major HMOS expected to be present in milks of respective milk types.

**Maternal genotype**		**Frequency in France/Europe (31)**	**Prominent HMOS expected in milk group**	**Milk group**
**Secretor**	**Lewis**			
Se/–	Le/–	69	2′-FL, 3-FL, DFL, LNT, LNnT, LNFP I, LNFP II, LNFP III LNDFH I, LNDFHII, 3′-SL, 6′-SL	Type I
se/se	Le/–	20	3-FL, LNT, LNnT, LNFP II, LNFP III, LNDFH II, 3′-SL, 6′-SL	Type II
Se/–	le/le	9	2′-FL, 3-FL, DFL, LNT, LNnT, LNFP I, LNFP III, 3′-SL, 6′-SL	Type III
se/se	le/le	1	3-FL, LNT, LNnT, LNFP III, 3′-SL, 6′-SL	Type IV

An active Se gen codes for FUT2 which transfers fucose via an α 1-2 glycosidic linkage. Prominent HMOS resulting from FUT2 activity are e.g., 2′-FL and LNFP I. Glycans like LNFP I which are carrying the reducing terminus Fuc (α1-2)Gal (β1-3)GlcNAc belong to the group of Le^d^ or H type 1 antigens. H type antigens link the Le/Se system with the blood group ABH system (31). In contrast, an active Le gene codes for FUT3 which in turn enables fucosylation via either α1-3 or α1-4 glycosidic linkage. FUT3 related structures are e.g., LNFP II and LNFP III. LNFP III is also an example for an Lewis^x^ (Le^x^) structural motif, whereas LNFP II represents a Lewis^a^ (Le^a^) epitope. Le^a^ epitopes are characterized by the carbohydrate sequence Gal (β1-3)[Fuc (α1-4)]GlcNAc-R. Le^x^-antigens contain type II structures with the following residue: Gal (β1-4)[Fuc (α1-3)]GlcNAc-R. If both, Se and Le genes are active, fucosylated HMOS structures bearing either one, two or all the possible types of fucosylation (i.e., via α 1-2, α 1-3, and α 1-4 glycosidic linkages) can occur. Lacto-N-difucohexaose I (LNDFH I) which also resembles a Lewis^b^ epitope with the monosaccharide motif Fuc (α1-2)Gal (β1-3)[Fuc (α1-4)]GlcNAc-R, is a known metabolite of joined FUT2 and FUT3 activity. It is noteworthy to mention that also other, Le/Se-system independent fucosyl-transferases may contribute to formation of α 1-3-fucosylated HMOS such as 3′-FL or LNFP V.

**Figure 2:**
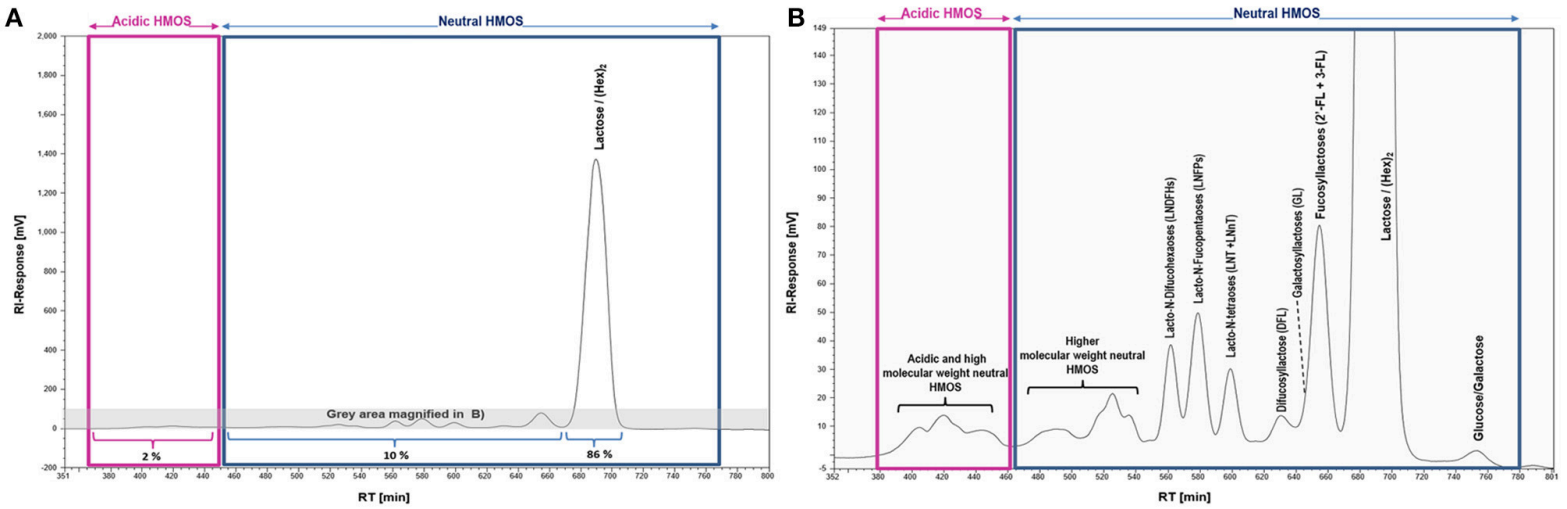
SEC-RI profiles of the total HMOS and mineral fraction from pooled human milk; **(A)** Full SEC-RI Profile, 86% of compounds detected by RI consist of Lactose/(Hex)_2_, 10% of other neutral HMOS and 2% of acidic HMOS; **(B)** Magnified section of **(A)** zooming into acidic and neutral HMOS; HM sampling, pooling, isolation of the total HMOS fraction and SEC-RI analysis have been performed as described earlier (32).

The complexity and relative abundance of different HMOS contained in human milk can for instance be characterized by size exclusion chromatography (SEC) and coupled refractive index detection (RI). A resulting SEC-RI trace is shown in Figure 2. Even more detailed information about complexity and individual monosaccharide compositions of HMOS could be derived by a subsequent MALDI-MS (33) analysis of individual SEC HMOS fractions. The acidic sub-fraction adds a further dimension to the overall variety of HMOS. The total number of neutral and acidic HMOS structures based on the MALDI-MS analyses of total human milk carbohydrate SEC-fractions is estimated to exceed the number of 1,000 different structures (24).

Based on the Le/Se status of the mother and specifically the related fucosylated HMOS structures found in the respective human milks, milk group systems of 4 different milk types have been defined (27). Therefore, it is possible to determine individual human milk types by probing presence of specific fucosylated HMOS like 2′-FL, DFL, LNFP I, LNFP II, LNDFH I, and LNDFH II with suited analytical means. An overview of the relationship between maternal Le and Se genotype and some major HMOS structures present in the respective milk types is given in Table 1. A recent review has summarized most of the qualitative and quantitative approaches to characterize the diversity of HMOS structures present within human milk (21).

## Biological functions of the different HMOS

**Figure 3:**
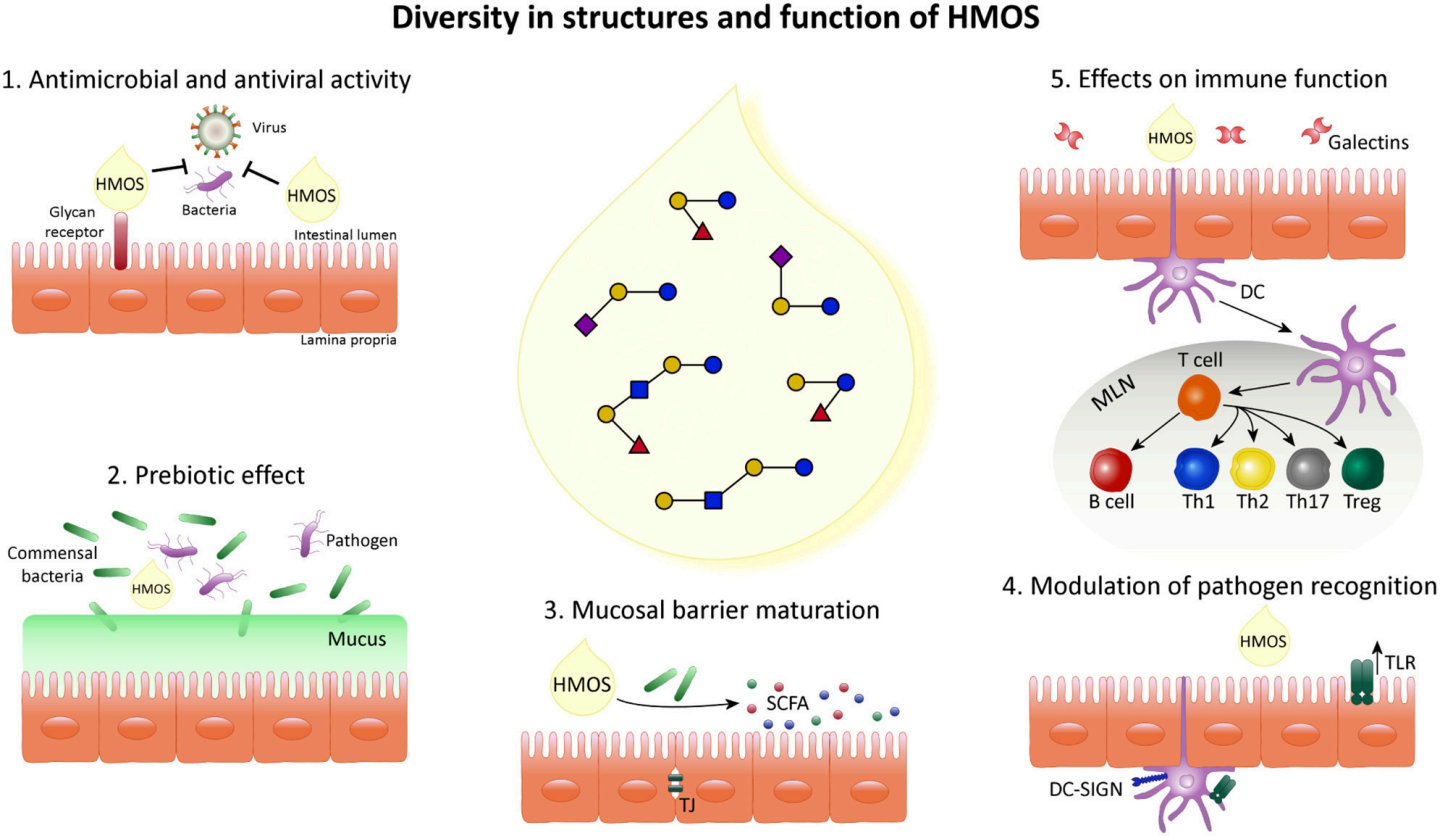
Schematic overview indicating the diversity in structure and function of HMOS. HMOS are composed of a complex mixture of oligosaccharides. This diversity of structures results in various roles in the epithelial cell layer, surrounding mucosa and immune system composing the digestive tract of breastfed infants. (1) HMOS have shown antimicrobial and antiviral effects by binding to virus bacteria, toxins and/or eukaryotes reaching the mucosal surfaces as well as by direct binding to epithelial surface receptors and blocking the access of pathogens. Thus, avoiding their replication and subsequent infection. (2) Commensal bacteria, illustrated as Bifidobacteria, metabolize HMOS and thus, their growth is promoted while pathogens less able to metabolize HMOS will experience growth suppression. (3) HMOS and Short Chain Fatty Acid (SCFA), metabolites of HMOS, were shown to influence intestinal epithelial cell (IEC) maturation by promoting differentiation while suppressing proliferation as well as tight junction development, required for proper intestinal barrier function. (4) Expression of receptors involved in pathogen recognition, such as TLR as well as their signal transduction was increased after HMOS exposure which in turn impacts the immune homeostasis. (5) DC in close proximity to the intestinal epithelial barrier are involved in the immunomodulatory effects described for HMOS. DCs exposed to HMOS play a role in the DC/T cell interaction leading to T cell differentiation and/or T cell/B cell interaction which may occur in secondary lymphoid organs, depicted as mesenteric lymph nodes (MLN), subsequently promoting immune homeostasis.

The presence of the unique diversity of HMOS, suggests different biological functions and mechanisms by which they may influence the infant's microbiome and immune maturation and their susceptibility to infections as summarized below and shown in Figure 3.

The topics exemplified in Figure 3 are further substantiated point by point in the following section.

### Antimicrobial and antiviral effects of HMOS

HMOS play a role in the prevention of infections in breastfed infants by direct blockage of viral and bacterial cellular pathogens and toxins infection by mimicking cell entry receptors (34–36). The first mechanisms by which HMOS may exert their anti-infective properties are through the inhibition of virus binding to the host cells by mimicking viral receptors and/or by blocking virus entry into the cell, as well as intracellularly, by blocking viral replication. The anti-infective potential of HMOS has been demonstrated for both neutral as well as acidic HMOS, for example different strains of Norovirus have affinity for specific HMOS structures (35, 37). In addition, both sialylated and fucosylated milk oligosaccharides reduced the infectivity of rotavirus (38). Interestingly, HMOS with multiple Le^x^ epitopes were shown to inhibit HIV-1 transfer to CD4+ T lymphocytes more efficiently than other HMOS structures (39). HMOS may also block microbial pathogen entry, since HMOS from pooled human milk were shown to significantly reduce *Escherichia coli* attachment to cultured epithelial cells (40). Likewise, it has been shown *in vitro* that LNT, or its fucosylated derivative LNFPI, both can inhibit the growth of Group B Streptococci (41). Moreover, the presence of 3′-FL within the complex mixture of HMOS structures has been inversely correlated with Group-B Streptococci abundancy in infants (42). In addition, α(1-2)-fucosylated HMOS like 2′-FL, or LNDFH I may reduce of early life diarrhea incidence and severity, via their ability to block specific diarrhea inducing pathogens (43).

### Prebiotic effect of HMOS

Development of selective bacterial strains is subjected to their capacity to metabolize HMOS (44). The role of microbial modulation i.e., the prebiotic capacity of specific HMOS structures have in addition been subject of extensive studies. More specifically, secretor positivity of mothers, hence expressing FUT2 and therefore able to produce α(1-2)-glycosidic-fucosylated HMOS, have been shown to affect the gut bifidobacterial communities of breastfed infants (45). Bifidobacteria and Bacteroides species are known to metabolize HMOS with high efficiency in contrast to other bacterial species such as *E. coli, Clostridia, Eubacteria, Enterococci* (44). This appears strain specific and selective for specific HMOS structure (44, 46, 47). For example, *Bifidobacteria* exhibited strong growth stimulation while expansion of *Clostridium perfringens* and *E. coli* were suppressed within cultures using specific HMOS (like 2′-FL, 3-FL, and LDFT), whereas Enterobacteria could not grow on 2′-FL or 6′-SL cultures (48). In addition, utilization of fucosylated type human milk oligosaccharides by isolated human gut microbes was shown (49). These data indicate selective and specific prebiotic capacities of different functional HMOS structures, showing growth of commensal bacteria such as *Bifidobacteria* at the expense of pathogens, as shown in Figure 3. Hence beyond directly blocking viral and bacterial entrance to the host also these prebiotic capacities of HMOS may help to reduce the susceptibility to infection of the host.

### Mucosal barrier maturation by HMOS

HMOS interact with glycans present in the surface of intestinal epithelial cells (IEC) or with dendritic cells (DC) which protrude to the gut lumen from lamina propria. This results in direct support of epithelial barrier maturation or an indirect effect on barrier integrity via modulation of the microbiota and consequent short chain fatty acid (SCFA) production (50). In this regard, beyond blocking pathogen invasion, HMOS may also promote mucosal barrier maturation by increasing the differentiation of IECs. Indeed, synthetic HMOS or HMOS isolated from human milk were shown to promote differentiation and reduce proliferation of various IEC cultures (HT-29 and Caco-2). Similarly, expression of mucosal maturation factors was promoted in fetal intestine cultures after exposure to HMOS isolated from colostrum. These findings suggest that some specific HMOS may be able to promote gut maturation and contribute to epithelial barrier integrity in the gastrointestinal tract of neonates (18, 50, 51).

### Modulation of pathogen recognition by HMOS

Receptors involved in the recognition of microbes such as toll-like receptors (TLR) are suggested to be modulated by HMOS. Subsequently the response of the host cell to pathogens is altered (17, 37). *In vitro* studies to elucidate the receptors involved in HMOS effects have been performed mostly in cells isolated from adult individuals which might not translate directly to the neonatal situation. Specific HMOS structures have been postulated to modulate bacterial and viral signaling on epithelial cells and/or DC (19). For instance, 2′-FL modulates CD14 expression in human enterocytes, thereby attenuating LPS-induced inflammation *in vitro* (17). On the contrary, HMOS such as sialyllactoses, human galactosyllactoses and/or LNFP III may be ligands for toll like receptors (TLR). For example, TLR-3 signaling seems specifically inhibited by human milk 3′-galactosylactose (52). Moreover, it has been shown that the addition of human milk as well as HMOS interacts directly with DCs, through DC-SIGN, Siglecs and related glycan-binding proteins which are also essential in immune regulation (53–55). DCs are key in directing the adaptive immune response toward effective immunity identification and clearance pathogens. Alpha-fucosylated HMOS (2′-FL and 3-FL) showed specific binding to DC-SIGN (54). Effects of scGOS/lcFOS were suggested to be mediated by TLR-4 (56). Similarly, TLR-4 as well as TLR-3 have also been related to modulate the effects of HMOS. 3′-FL, 2′-FL were able to modulate TLR-3 and elicit an anti-inflammatory effect, while exposure to 2′-FL inhibited inflammation through TLR-4 (52). More specifically it has clearly been shown that the addition of scGOS/lcFOS ameliorates the microbial composition reducing the presence of clinically relevant pathogens (57). Selectins were also suggested as possible receptors for binding of HMOS due to their ability to block P-selectin (58). Several receptors are hypothesized to be involved in the recognition of HMOS. The diversity of HMOS structures present in human milk might determine HMOS-glycan receptor binding. HMOS target TLRs and C-type lectins which are vital in pathogen recognition, immune modulation and essential during development of the immune system in early life. Therefore, HMOS may contribute to the development of a balanced and effective immune response, hereby providing protection toward infections.

### Effect on immune system development by HMOS

Specific HMOS, such as 2′-FL, 3′-SL, 6′-SL, and LNT have been detected within the intestine as well as in systemic circulation of breastfed infants (23, 59, 60). Increasing evidence collected during the past two decades suggesting a role of HMOS directly on immune cells. Despite all efforts, the effects described remain rather incomplete (19). Nevertheless, it is suggested that HMOS play a role in supporting the developing mucosal and systemic immune system (13, 16). HMOS derived from human colostrum can modulate intrinsic expression of inflammatory markers associated with cell trafficking and modulate signaling pathways related to maturation of lymphoid tissue and influence cytokine and chemokine networks that regulate Th1/Th2 lymphocyte balance. The anti-inflammatory effect of for instance 2′-FL is known. 2′-FL from pooled human milk showed the ability to dampen pro-inflammatory mediator IL-8 release from T84 IEC line after type 1 pili *E. coli* infection (17). Similarly, reduced IL-8 expression was measured in fetal intestinal human tissue when exposed to 3′-, 4- and 6′-galactosyllactoses from human milk colostrum (17, 52). 2′-FL was shown to inhibit the inflammatory mediators secreted after TNFα induced *in vitro*, possibly through the inhibition of NF-κB activation (61). Furthermore, *in vitro* data demonstrate 2′-FL and LNFP I to be able to reduce monocyte activation and to modulate the release of IFNγ, IL-12, and IL-10 (62).

In addition, specific prebiotic oligosaccharides have been demonstrated to be immune modulatory (63–65). Immunomodulatory effects have been demonstrated for 2′-FL, suggesting an additional function of specific oligosaccharides (66–68). However, if these effects also relate to improved infection susceptibility in infants remains to be established. From the *in vitro* based human milk immune cell interaction studies some specific anti-inflammatory effects have been identified.

Galectins are another class of lectins involved in the regulation of immune and inflammatory processes (55). Interestingly, HMOS are reported to bind to various recombinant human galectins like hGal-1, -3, -4, -7, -8, and -9 in a very structure dependent and selective way. Human milk glycans with terminal type I sequences (Galβ1-3 GlcNAc) preferentially bind to hGal-7, whereas hGal-2 did not bind to human milk glyco-types but to a human blood group A Type 2 determinant (55). Beyond serving as a glycan receptor, galectins can also be secreted as soluble mediators and affect immune function. In this regard, IEC derived galectin-9 was increased after exposure of IEC to a mixture of scGOS/lcFOS in combination with a TLR-9 ligand in an *in vitro* co-culture model of IEC and activated immune cells (69). Galectin-9 played a key role in enhancing IFNγ and IL-10 production by immune cells underlying the IEC in this model (55). Further research will reveal the specific role of galectins in immunomodulation after exposure to HMOS, as well as their similarities with the immunomodulatory properties seen by scGOS/lcFOS. However, it is important to realize that an efficient immune response remains to be mounted against the intruding pathogen. Providing efficient protection, in most cases, will go hand in hand with the induction of inflammation. If an anti-inflammatory response is beneficial in relation to the protection against pathogens, will be pathogen and host specific, and can only be elucidated *in vivo*.

## Human milk oligosaccharide impact *in vivo*

It is the unique complexity of human milk oligosaccharides which leads to the speculation that these abundantly available structures in human milk play a key role in providing protection against infections in neonates. From the limited *in vivo* studies, we know that specific HMOS structures can reduce the interaction of specific pathogens like *Salmonella, Shigella, Vibrio cholerae, E. coli*, Polioviruses, Rotavirus and Respiratory Syncytial virus (RSV) with the host (11, 70). Within some studies, levels of 2′-FL, lacto-N-difucohexaose (LNDFH I), (α 2-linked fucosyloligosaccharide) and ratios between 2-linked and 3-/4-linked oligosaccharide were associated, with presence of specific pathogens like *E. coli, Campylobacter* and *Norovirus* (35, 43). Interestingly, the provision of secretory type related complex mixtures of HMOS, have been associated with a direct protection against specific infections (71). Fucosyltransferase 2 non-secretor and low secretor status seems to associate with severe outcomes in premature infants. Meaning that within this study a low secretor phenotype was associated with the onset of NEC, and non-secretor genotype was associated with gram negative sepsis (71). In addition, it has been suggested that FUT2, the regulator of Lewis and ABO(H) antigens in the intestinal mucosa, could be a host genotypic feature affecting susceptibility to ETEC infection (72). Several intervention studies have reported the functional benefit of adding prebiotic oligosaccharides to infant formula. More specifically, specific prebiotic oligosaccharides have been shown to ameliorate the development of allergies as well as reduce the impact of pediatric infections (4, 5, 73–75). In this regard, the immune modulating effect that seems to decrease the risk on developing atopy and allergy, also may lower the infection risk in neonates, which is suggestive for basic immune modulation early in life. The clinical consequences of specific individual HMOS structures however, remain to be further elucidated (76). The first clinical safety studies are now reported on the use of specific HMOS combinations i.e., 2′-FL and scGOS (60) or the combination of two single oligosaccharides 2′-FL and LNnT (68, 77). Although growth and 2′-FL uptake were similar between formula receiving infants and as seen in breastfed infants, the possible functional benefits regarding immune development and/or infection susceptibility related to a single oligosaccharide are however not extractable from these studies. Therefore, the identification and understanding of protective elements in human breast milk decreasing infant's susceptibility to infection remains limited (75).

In conclusion, components of breast milk (including HMOS) play a key role in the development of the neonatal immune system by preventing pathogen replication, promoting healthy microbial diversity, inducing maturation of intestinal mucosa and by modulation of immune cells as well as pathogen recognition receptors. Currently, there is little understanding about the role of the diverse HMOS structures in optimally inducing microbiome and immune development and consequently how they may provide protection against infections. Therefore, we postulate that HMOS are involved in regulation of mucosal immune and barrier function in multiple ways, although the specific mechanisms remain poorly understood and may be a compilation of the biological functions of individual structures and their interactions. Further investigation into the components of breast milk and their roles in providing protection to infants is required, irrespective of the mechanism by which specific HMOS structures can provide protection toward certain pathogens.

## Author contributions

VA-M, AvS, and MM have written the review. LW, JG, and BvL supervised the program. BS made specific contributions to the program with regard to human milk and in particular functional oligosaccharides. All authors listed have approved it for publication.

### Conflict of interest statement

JG is head of the Division of Pharmacology, Utrecht Institute for Pharmaceutical Sciences, Faculty of Science at the Utrecht University and partly employed by Nutricia Research. BvL is leading a strategic alliance between University Medical Centre Utrecht/Wilhelmina Children's Hospital and Nutricia Research. BS, MM, and BvL are employed by Nutricia Research. The remaining authors declare that the research was conducted in the absence of any commercial or financial relationships that could be construed as a potential conflict of interest.

## Abbreviations

2′-FL: 2′-Fucosyllactose
3′-SL: 3′-Sialyllactose
6′-SL: 6′-Sialyllactose
APC: Antigen-presenting cells
DC: Dendritic cell
DC-SIGN: Dendritic Cell-Specific Intercellular adhesion molecule-3-Grabbing Non-integrin
FoxP3: Forkhead box protein 3
FUT 2: Fucosyltransferase 2
HMOS: Human Milk Oligosaccharides
lcFOS: Long Chain Fructo-oligosaccharides
MHC-I (II): Major Histocompatibility Complex Class I (II) molecules
SCFAs: Short Chain Fatty Acids
scGOS: Short Chain Galacto-oligosaccharides
tDC: tolerogenic dendritic cells
Th: T-helper cell
TJ: Tight-Junction
TLRs: Toll-Like Receptors
Tregs: Regulatory T cells.
